# Comparison of cytotoxicity between extracts of *Clinacanthus nutans* (Burm. f.) Lindau leaves from different locations and the induction of apoptosis by the crude methanol leaf extract in D24 human melanoma cells

**DOI:** 10.1186/s12906-016-1348-x

**Published:** 2016-09-20

**Authors:** Siat Yee Fong, Terrence Piva, Chaitali Dekiwadia, Sylvia Urban, Tien Huynh

**Affiliations:** 1 Faculty of Medicine and Health Sciences, Universiti Malaysia Sabah, Jalan UMS, 88400 Kota Kinabalu, Sabah Malaysia; 2 School of Health and Biomedical Sciences, RMIT University, PO Box 71, Bundoora, 3083 VIC Australia; 3 RMIT Microscopy and Microanalysis Facility, RMIT University, GPO Box 2476, Melbourne, 3001 VIC Australia; 4 School of Science, RMIT University, GPO Box 2476, Melbourne, 3001 VIC Australia; 5 School of Science, RMIT University, PO Box 71, Bundoora, 3083 VIC Australia

**Keywords:** *Clinacanthus nutans*, Melanoma, Anticancer, Cytotoxic, Apoptosis

## Abstract

**Background:**

*Clinacanthus nutans* (Burm. f.) Lindau leaves are widely used by cancer patients and the leaf extracts possess cytotoxic and antiproliferative effects on several human cancer cell lines. However, the effect of *C. nutans* leaf extract on human melanoma, which is the least common but most fatal form of skin cancer and one of the most common cancers diagnosed in both sexes worldwide, is unknown. There is also limited information on whether the bioactivity of extracts differs between *C. nutans* leaves grown in different geographical locations with varying environmental conditions.

**Methods:**

The present study, for the first time, compared and demonstrated the cytotoxicity of the crude methanol extracts of *C. nutans* leaves from 11 different locations in Malaysia, Thailand and Vietnam, with diverse environmental conditions against D24 melanoma cells through WST-8 assay. The percentage of apoptotic cells following treatment with the most active extract was determined in a dose- and time-dependent manner by a cytofluorometric double staining technique. Biochemical and morphological changes in the treated and untreated cells were examined by fluorescence and transmission electron microscopy techniques, respectively, to further affirm the induction of apoptosis.

**Results:**

The leaves of plants grown at higher elevations and lower air temperatures were more cytotoxic to the D24 melanoma cells than those grown at lower elevations and higher air temperatures, with the leaf extract from Chiang Dao, Chiang Mai, Thailand exhibited the highest cytotoxicity (24 h EC_50_: 0.95 mg/mL and 72 h EC_50_: 0.77 mg/mL). This most active crude extract induced apoptotic cell death in the D24 cells in a dose- and time-dependent manner. Typical biochemical and morphological characteristics of apoptosis were also observed in the treated D24 cells.

**Conclusions:**

The results, showing the cytotoxicity of *C. nutans* and the induction of apoptosis in D24 cells, are significant and useful to facilitate the development of *C. nutans* as a potential novel chemotherapeutic agent for the management of skin melanoma.

**Electronic supplementary material:**

The online version of this article (doi:10.1186/s12906-016-1348-x) contains supplementary material, which is available to authorized users.

## Background

Cancer remains the leading cause of morbidity and mortality worldwide, with an estimated 14 million new cases and 8.2 million cancer related deaths in 2012. The number of new cases is predicted to rise by ~ 70 % over the next 20 years [1]. Melanoma is a type of skin cancer, characterised as a neoplastic disorder of the epidermal pigment-producing cells known as melanocytes [2]. Melanoma is the least common but most fatal form of skin cancer and it has increased two-fold in the past two decades [3]. It is one of the most common cancers diagnosed in men and women worldwide, where 232,130 incidences were estimated in 2012 [4]. Although melanoma can be surgically treated at early stages, it is an aggressive tumour with advanced disease defined by widespread metastatic lesions and the tumour has been reported to be resistant to most forms of cancer treatment [5]. Therefore, it has become an increasingly critical public health concern and novel treatment options are urgently required.

Besides surgery, cytotoxic chemotherapeutic drugs have also been used to treat melanoma [6]. However, these drugs can cause severe adverse effects and multi-drug resistance [7–9], which remain a major dilemma to many cancer patients. Recently, there has been an increasing interest in the areas of natural products for novel and bioactive molecules for cancer drug discovery due to their general availability, safety and low toxicity, which may cause lesser side effects [10]. Phytochemicals from roots, bulbs, barks, stems, flowers and leaves have been shown to have anticancer property [11]. These compounds can be potential sources of anticancer agents and new drug synthesis [12].

*Clinacanthus nutans* (Burm. f.) Lindau is a medicinal plant native to Southeast Asia with reported bioactivities, such as anti-inflammatory [13], antioxidant [14–18], antidiabetic [18], antimicrobial [16] and antiviral against herpes simplex virus (HSV) type 1 [19–21] and 2 [21, 22], varicella-zoster virus (VZV) [23], human papillomavirus (HPV) [24] and dengue virus [25]. Moreover, *C. nutans* leaves also possess antiproliferative effects on human erythroleukemia (K-562), Burkitt’s lymphoma (Raji) and cervical carcinoma (HeLa) cells [15, 16]. However, the cytotoxicity of *C. nutans* leaf extract against melanoma cells, how it induces cell death as well as the effect of collection sites are still unknown. Therefore, the current study aimed to i) investigate and compare the cytotoxicity of the crude methanol extracts of *C. nutans* leaves collected from 11 different locations with varying environmental characteristics against the D24 melanoma cells, ii) evaluate the cytotoxic effect and selectivity of the extract against the D24 cells in a dose- and time-dependent manner and iii) examine the possible death mode of the D24 cells induced by the extract using biochemical and microscopy techniques.

## Methods

### Plant materials

Fresh leaves of 11 *C. nutans* samples grown under different environmental conditions were collected from Peninsular Malaysia (CP), East Malaysia (CE), Thailand (CT), and Vietnam (CV1) (Table 1). Geographic data, including elevation, annual temperature (high, low and mean) and rainfall of sampling sites was obtained from DIVA-GIS version 7.5 software [26]. Prior to sample extraction, all leaf pieces were thoroughly washed using cold tap water. All samples were air dried in the shade for seven days at 22 °C and stored as the whole leaf in air-tight bags in darkness at 22 °C until further analysis. Samples were identified by Mr Julius Kulip at Biology Tropical and Conservation Institute, Universiti Malaysia Sabah and deposited in Borneensis Herbarium, Universiti Malaysia Sabah (voucher no. BORH 2093).

**Table 1:** The sample codes, collection sites and environmental conditions of collected samples

Sample	Country	State/Province	Region	Environmental conditions			
					Tm (°C)		
				Elv (m)	H	L	Rn (mm)
CP2	Malaysia	Negeri Sembilan	Seremban	83.4	31.2	22.4	2010.0
CE1	Malaysia	Sabah	Sandakan	158.7	30.8	22.9	2973.0
CE2	Malaysia	Sabah	Sandakan	74.9	30.8	22.9	2973.0
CE3	Malaysia	Sabah	Tawau	6.8	30.7	23.2	1975.0
CE4	Malaysia	Sabah	Kota Kinabalu	9.7	30.7	23.3	2818.0
CT1	Thailand	Nakhon Pathom	Map Khae	8.1	32.7	22.7	1237.0
CT2	Thailand	Nakhon Pathom	Map Khae	8.1	32.7	22.7	1237.0
CT3	Thailand	Nakhon Pathom	Salaya	4.3	32.5	23.4	1334.0
CT4	Thailand	Chiang Mai	San Sai	309.5	31.5	19.6	1191.0
CT5	Thailand	Chiang Mai	Chiang Dao	439.4	30.6	18.7	1261.0
CV1	Vietnam	Ho Chi Minh	Ho Chi Minh	2.2	31.9	23.1	1873.0

Abbreviation: *Elv* elevation; *Tm* mean annual temperature; *H* highest; *L* lowest; *Rn* mean annual rainfall

### Preparation of crude extracts

A preliminary study on the total phenolic and flavonoid content of the crude dicholoromethane, ethanol and methanol extracts of *C. nutans* leaves showed that the methanol extract had the highest levels of both phenolics and flavonoids. Therefore, methanol was chosen as the extraction solvent for the current study (Additional file 1: Table S1). One gram of dried powdered *C. nutans* leaves from each location was extracted with 50 mL of methanol (MeOH) (Merck, Germany), on an orbital shaker at a speed of 200 rpm at 22 °C for seven days. The extracted solution was decanted, filtered with Whatman No. 1 filter paper and concentrated under reduced pressure using a rotary evaporator (Buchi, Switzerland) to produce the crude MeOH dried extract, which was then stored at −20 °C for further analyses.

### Cell line and culture conditions

D24 melanoma cells and human dermal fibroblasts (NHDF) were cultured in RPMI 1640 medium containing L-glutamine (Gibco, Life Technologies, USA) and high-glucose DMEM medium with pyruvate and L-glutamine (Gibco), respectively, supplemented with 10 % (v/v) FBS (Serana, Australia) and 1 % (v/v) penicillin/streptomycin (Gibco). For all the experiments, the cells were incubated for the indicated time under the indicated treatment at 37 °C in a humidified atmosphere of 5 % CO_2_.

### Cytotoxicity assay

The cytotoxicity of the crude MeOH leaf extract of 11 *C. nutans* samples was measured using the Cell Counting Kit-8 (CCK-8) (Sigma-Aldrich, USA) according to the manufacturer’s instructions. Briefly, the D24 and NHDF cells were seeded at 5 × 10^3^ cells/well in 96-well, flat bottomed plates (Greiner, Austria). After 24 h, the cells were treated with the different extracts at 2 mg/mL prepared in DMSO (Sigma-Aldrich) with a final concentration of < 0.1 % (v/v) and incubated for 72 h. Cytotoxicity was measured at 450 nm using a microplate reader (CLARIOstar, BMG Labtech, Germany). The percentage of viable cells was determined relative to the vehicle control (<0.1 % DMSO) using the following equation:

$$ Viable\  cell\ \left(\%\right)=\left( absorbance\  of\  sample/ absorbance\  of\  control\right)\times 100 $$

The vehicle control was expressed as 100 %. Only the most active *C. nutans* sample (CT5) against the D24 cells as determined in this assay were used for further experiments.

### Determination of half maximal effective concentration (EC_50_)

The D24 and NHDF cells were seeded in 96-well plates as described in the previous section. The cells were treated with different concentrations (0–2 mg/mL) of the crude CT5 MeOH leaf extract and incubated at 24 and 72 h. At the end of each period, the percentage of viable cells was determined using the CCK-8 method (cytotoxicity assay). The percentage of viable cells was determined relative to the vehicle control cells (100 %). The EC_50_ values were determined from a non-linear regression model (curvefit) based on the sigmoidal dose–response curve (variable) and computed using GraphPad Prism version 6.05 (GraphPad Software, Inc., San Diego, USA).

### Observation of D24 cell morphology by phase contrast microscopy

Morphology of the untreated (control) D24 cells and cells treated with 1 or 2 mg/mL of the MeOH extract for 24 and 72 h, in 96-well plates (Greiner), were observed using a Nikon Eclipse TS100 (Japan) inverted microscope under 20× objective. Images were captured with the DS-Fi 1 camera and DS-L2 control unit.

### Evaluation of apoptosis by Muse cytofluorometric analysis

Double staining with Annexin-7 and 7-AAD was performed using the Muse Annexin V/Dead Cell Assay Kit (Merck Millipore, Germany). The untreated and treated D24 cells with 1 or 2 mg/mL of the MeOH extract for 24 and 72 h were harvested and resuspended in 100 μL of tissue culture medium. Then, 100 μL of the fluorescent reagent was added to the cell suspension and incubated for 20 min at 22 °C in the dark before being analysed for the detection of early and late apoptotic cells using a Muse Cell Analyzer (Merck Millipore). Based on the positivity of Annexin V, corresponding to phosphatidylserine externalisation in apoptotic cells and simultaneous detection of dead cells, positive for the nuclear dye 7-AAD, the assay allows the differentiation of four populations in each sample by cytofluorometric separation on a Muse Cell Analyzer (Merck Millipore): i) viable (lower left quadrant: Annexin V^−^/7-AAD^−^), ii) early apoptotic (lower right quadrant: Annexin V^+^/7-AAD^−^), iii) late apoptotic/necrotic (upper right quadrant: Annexin V^+^/7-AAD^+^) and iv) cell debris (upper left quadrant: Annexin V^−^/7-AAD^+^) cells.

### Detection of apoptosis and necrosis by Annexin V/PI double staining and confocal microscopy

The early and late apoptotic D24 cells were detected using the Annexin V-FITC kit (Beckman Coulter, USA), according to the manufacturer’s instructions with slight modifications. After treating the D24 cells with 1 or 2 mg/mL of the MeOH extract for 72 h, the cultures were stained with 1 μL of Annexin V-FITC (0.25 μg/mL) for 15 min, followed by 0.5 μL of PI (0.125 μg/mL) for 5 min in the dark. The treated and untreated cells were then observed using an inverted confocal microscope (Nikon Eclipse Ti-E A1, Japan) under 40× objective. The excitation wavelengths for Annexin V-FITC and PI used were 488 and 536 nm, respectively, while the emission wavelengths were 525 and 617 nm, respectively. Based on the principles of this technique, the normal cells would not be stained by the two dyes (Annexin V-FITC^−^/PI^−^); the early apoptotic cells would only be dyed by Annexin V-FITC (Annexin V-FITC^+^/PI^−^); the late apoptotic cells would be positive in both Annexin V-FITC and PI staining (Annexin V-FITC^+^/PI^+^).

### Ultra structural analysis by transmission electron microscopy (TEM)

The D24 melanoma cells were treated with the MeOH extract at 2 mg/mL for 72 h. Subsequently, the treated and untreated cells were harvested and resuspended in 100 μL of 2.5 % (v/v) glutaraldehyde with 2 % (w/v) paraformaldehyde in 0.1 M sodium cacodylate buffer, pH 7.3 at 22 °C for 30 min. Cells were then pelleted at 800 × g for 5 min and rinsed with 100 μL of 0.1 M sodium cacodylate buffer for 5 min and repeated three times. The cells were fixed in 100 μL of 1 % (w/v) osmium tetroxide with 1.5 % (w/v) potassium ferrocyanide at 22 °C for 1 h on an orbital shaker. After the period, the cells were centrifuged at 800 × g for 5 min and the supernatant was discarded. Then, the cells were washed by resuspending them in 100 μL of distilled water and were left on the orbital shaker for 10 min before centrifugation at 800 × g for 5 min. This step was repeated twice. Dehydration was performed as follows: the cells were first resuspended with 100 μL of 50 % (v/v) ethanol and left on an orbital shaker for 15 min before centrifugation at 800 × g for 5 min. The supernatant was discarded and followed by resuspension in 100 μL of 70 % (v/v) ethanol for 15 min, 100 μL of 90 % (v/v) ethanol for 15 min, 100 μL of 95 % (v/v) ethanol for 15 min, 100 μL of 100 % (v/v) ethanol for 30 min twice and finally 100 μL of 100 % (v/v) acetone for 30 min twice. Infiltration was carried out twice with the acetone: Spurr’s resin (1:1) mixture on a rotator, first overnight and then for 2 h at 22 °C. This was followed by vacuum infiltration with fresh 100 % Spurr’s resin for 2 h, repeated twice. Finally, the cells were polymerised at 70 °C for 24 h. The cells were sectioned to a thickness of 1 μm using a UCT ultramicrotome (Leica Ultracut, Germany). The sections were observed at 80 kV with a JEOL JEM 1010 (Japan) transmission electron microscope and images were examined using the Gatan Microscopy Suite software version 2.3 [27].

### Statistical analysis

All assays and cell experiments were performed in triplicate, unless otherwise indicated and the results presented as mean ± SD. Data was analysed using statistical software Minitab 17 [28]. Significant difference was determined using ANOVA Fisher’s test at *p* ≤ 0.05 significance level.

## Results

### Comparison of cytotoxicity of the crude methanol extracts of *C. nutans* leaves obtained from different locations against the D24 melanoma cells

There were differences in levels of cytotoxicity of the MeOH extracts (2 mg/mL) of *C. nutans* leaves from 11 different locations against the D24 cells after 72 h exposure (Table 2). All the extracts except for CT1 and CT2, were significantly (*p* ≤ 0.05) cytotoxic compared to the vehicle control (<0.1 % DMSO). Cytotoxicity of all extracts varied from 19.8 to 91.0 %, with a comparative mean effect of 52.5 ± 25.2 %. CT5 from Chiang Dao exhibited the highest activity, which was 5-fold greater than that of CT2 from Map Khae with the lowest activity. Of the 11 *C. nutans* samples studied, only CE1, CE4, CT4, CT5 and CV1 at 2 mg/mL caused > 50 % cytotoxicity.

**Table 2:** Effect of the crude MeOH leaf extract of *C. nutans* samples from 11 different locations at 2 mg/mL on the D24 cells after 72 h exposure as determined by WST-8

Sample	Cell viability (% of control)
	Mean ± SD (n = 3)
CP2	55.02 ± 10.95*
CE1	48.49 ± 9.39*
CE2	70.45 ± 11.32*
CE3	62.60 ± 10.48*
CE4	27.22 ± 6.24*
CT1	79.79 ± 14.10
CT2	80.16 ± 15.76
CT3	50.35 ± 13.75*
CT4	17.71 ± 6.25*
CT5	9.01 ± 4.62*
CV1	22.10 ± 3.95*

Note: * *p* ≤ 0.05, significantly different from vehicle control (<0.1 % DMSO)

### Cytotoxicity of the crude methanol *C. nutans* leaf extract against the D24 melanoma and NHDF cells

**Fig. 1:**
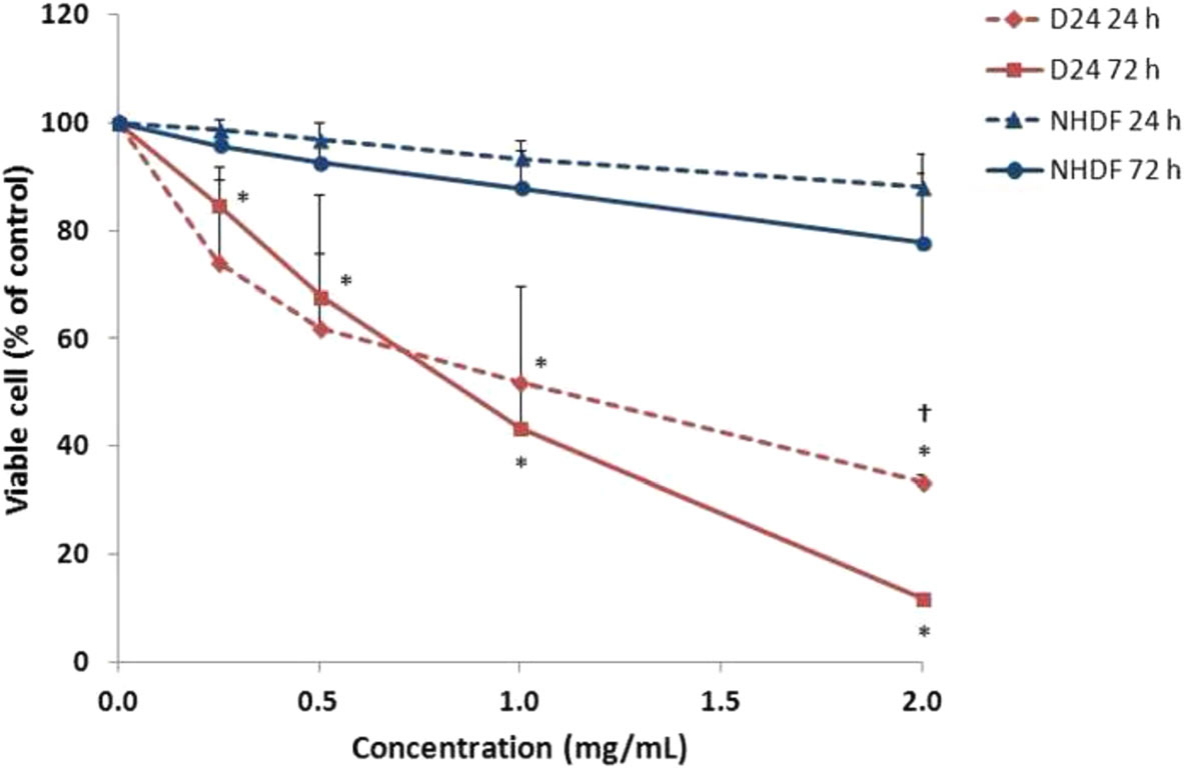
Cytotoxic effect of the crude MeOH *C. nutans* leaf extract on the D24 melanoma and NHDF cells. The cells were treated with different concentrations (0–2 mg/mL) of the extract for 24 and 72 h. Data represents mean ± SD from two independent experiments (*n* = 2). * *p* ≤ 0.05, significantly different from vehicle control (<0.1 % DMSO). † *p* ≤ 0.05, significantly different between treatment times

It was observed that the MeOH extract of CT5 was the most cytotoxic. Hence, this sample was selected for further experiments, including determination of the EC_50_ for the leaf extract. After 24 h exposure, the MeOH extract at 1 and 2 mg/mL showed significant (*p* ≤ 0.05) cytotoxicity against the D24 melanoma cells, which caused 66.5 and 48.0 % cell death, respectively, compared to the vehicle control (<0.1 % DMSO) (Fig. 1). Viability of the D24 cells fell sharply after exposure for 24 h to lower concentrations (0.25 and 0.5 mg/mL) of the extract, even lower than those exposed for 72 h. However, increasing the extract concentration above 1 mg/mL showed a more gradual reduction of viable cells.

Meanwhile, all test concentrations of the MeOH extract significantly (*p* ≤ 0.05) reduced the percentage of viable D24 cells after 72 h exposure, compared to the vehicle control (<0.1 % DMSO) (Fig. 1). Treatment with 2 mg/mL of the extract caused 88.0 % cell death, almost 6-fold more cytotoxic than that with 0.25 mg/mL (15.3 % cell death). Furthermore, there was a significant (p ≤ 0.05) difference in cytotoxic effect between 24 and 72 h at 2 mg/mL, suggesting that the observed effect was time dependent. The EC_50_ values for 24 and 72 h were 0.95 and 0.77 mg/mL, respectively.

Also shown in Fig. 1, the MeOH extract showed low cytotoxicity against the normal NHDF cells, where 24 and 72 h exposure to 2 mg/mL of the extract caused 12.0 and 22.2 % cell death, respectively. The EC_50_ values for the extract at 24 and 72 h were > 2 mg/mL, suggesting that the extract was more selective for the D24 melanoma cells than the normal cells.

### Morphological changes in the D24 melanoma cells treated with the crude methanol *C. nutans* leaf extract

**Fig. 2:**
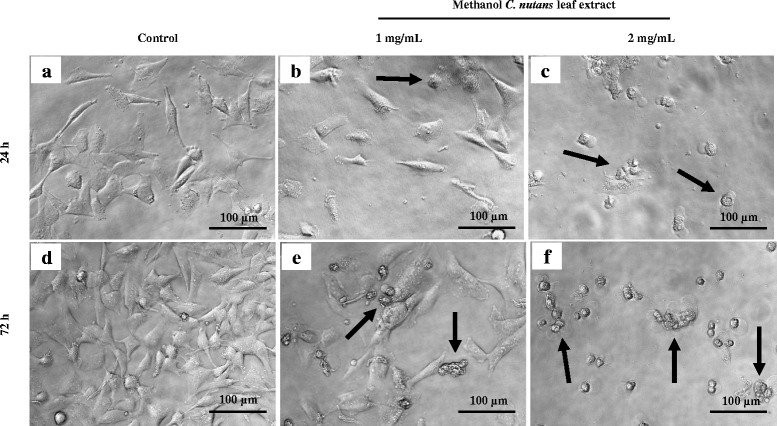
Morphological changes (arrows) in the D24 melanoma cells treated with the crude MeOH *C. nutans* leaf extract. Phase contrast images (20×) of cells after 24 h exposure to no treatment (**a**), extract at 1 mg/mL (**b**) and 2 mg/mL (**c**); 72 h exposure to no treatment (**d**), extract at 1 mg/mL (**e**) and 2 mg/mL (**f**)

The untreated (control) D24 melanoma cells were adherent and exhibited a smooth surface and elongated structure. These cells also showed a finely granulated cytoplasm (Fig. 2a & d). In contrast, the cells treated with the extract displayed notable cell shrinkage with irregular and rough form and the cell number were lower than that of controls (Fig. 2b, c, e & f). More noticeable morphological changes were observed when the cells were exposed for longer treatment times and at higher concentrations, which corresponded to the results of the cytotoxicity assay.

### Induction of mode of cell death in the treated D24 melanoma cells by the crude methanol *C. nutans* leaf extract

**Fig. 3:**
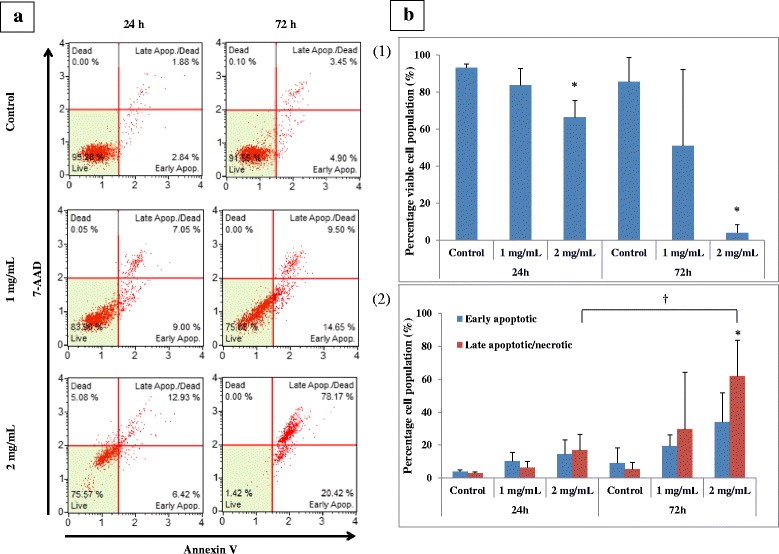
Effect of the crude MeOH *C. nutans* leaf extract on the D24 melanoma cells. Representative apoptosis profile plots of the untreated and treated cells after 24 or 72 h exposure to 1 or 2 mg/mL of the extract (**a**). The percentage of viable (**b**1), early apoptotic and late apoptotic/necrotic (**b**2) cell populations of untreated and treated groups. Data represents mean ± SD from three independent experiments (*n* = 3). * *p* ≤ 0.05, significantly different from control. † *p* ≤ 0.05, significantly different between treatment times

Treatment of the D24 cells with the extract at 1 mg/mL for either 24 or 72 h did not induce high affinity for both Annexin V and 7-AAD, indicating that most cells (>50 %) were viable (bottom left quadrant) (Fig. 3a & b1). Similarly, 24 h exposure to the extract at 2 mg/mL also showed that most of the D24 cells were viable. However, treatment with 2 mg/mL of the extract for 72 h remarkably increased the number of cells that were positive to both Annexin V and 7-AAD (upper right quadrant). This was also shown in Fig. 3b2. When the cells were treated with 2 mg/mL of the extract for 72 h, there was a significant (*p* ≤ 0.05) increase in the percentage of late apoptotic/necrotic cells compared to the control. Besides, when the cells were exposed to 2 mg/mL of the leaf extract for 72 h, the number of cells that had undergone apoptosis increased by almost 4-fold than those exposed for 24 h. This indicated that a long exposure to a high concentration of the MeOH extract was more likely to induce late apoptosis/necrosis rather than early apoptosis in the D24 cells.

**Fig. 4:**
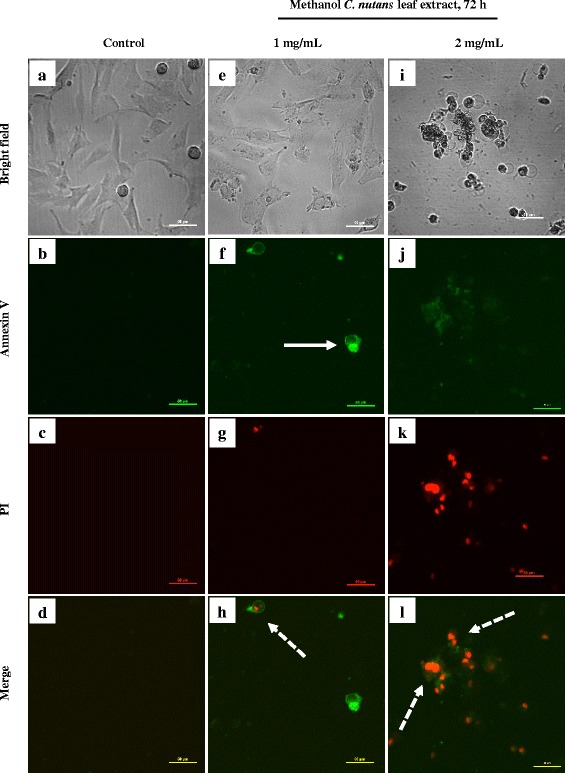
Confocal images (40 ×) of the untreated cells with no uptake of any stains (**a**–**d**) and the induction of early and late apoptosis/necrosis in the D24 cells after treatment with 1 mg/mL (**e–h**) and 2 mg/mL (i-l) of crude MeOH *C. nutans* leaf extract for 72 h. Continuous arrow indicate early apoptotic cells and dotted arrows indicate late apoptotic/necrotic cells

Confocal microscopy was used to provide a qualitative identification of both apoptotic and necrotic deaths of the D24 cells treated for 72 h with 1 and 2 mg/mL of the extract (Fig. 4). The control (untreated) cells (Fig. 4a-d) did not stain following the addition of both Annexin V and PI, which showed that they were viable. Most of the D24 cells treated with 1 mg/mL of the extract were also negative to both fluorescent stains (Fig. 4e-h). However, early apoptosis (Annexin V + ve; PI -ve) (Fig. 4f) as well as late apoptosis/necrosis (Annexin V + ve; PI + ve) (Fig. 4h) were seen in several of the D24 cells treated with 1 mg/mL of the extract. On the other hand, most of the cells treated with the higher test concentration (2 mg/mL) were positive to both Annexin V and PI, which appeared red (Fig. 4l), indicating the presence of late apoptotic/necrotic cells. The cells treated with 2 mg/mL of the extract had a higher level of these fluorescent stains than compared to those treated with 1 mg/mL, which suggests that the modes of D24 cell death were dose-dependent. The results obtained from confocal microscopy were similar to that obtained from the cytofluorometric analysis.

### Ultrastructural changes in the D24 melanoma cells treated with the crude methanol *C. nutans* leaf extract

**Fig. 5:**
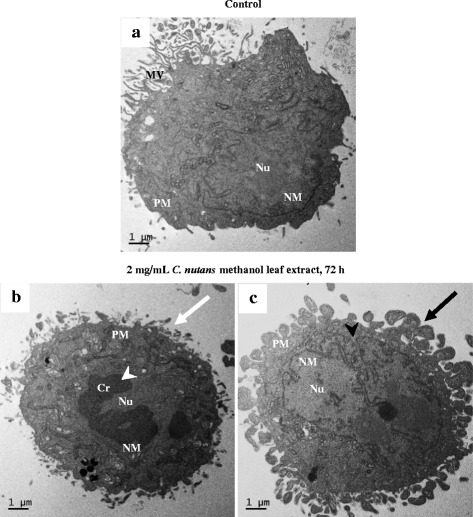
Transmission electron micrographs of the control (untreated) D24 cell (**a**) and cells treated with the crude MeOH extract (2 mg/mL) for 72 h (**b & c**). Distinct morphological changes, including plasma membrane alteration (white arrow), chromatin condensation (white arrowhead), blebbing (black arrow) and segmented/lobulated nucleus (black arrowhead) were observed in the treated cells. Cr: chromatin, MV: microvilli, NM: nuclear membrane, Nu: nucleus, PM: plasma membrane

As the crude MeOH *C. nutans* leaf extract was shown to induce cell death in the D24 cells, morphological changes in the cells were further analysed by electron microscopy to detect more details. In the D24 cells, clear morphological changes were observed between the untreated control and cells treated with the extract (2 mg/mL) for 72 h (Fig. 5). The untreated cells displayed of normal cell characteristics, such as microvilli, intact plasma membrane and nucleus with evenly distributed chromatin (Fig. 5a). In contrast, in the treated cells, there was cell shrinkage, loss of microvilli, marked peripheral chromatin condensation at the nuclear membrane (Fig. 5b), segmented/lobulated nucleus and irregular plasma membrane with extensive blebbing (Fig. 5c).

## Discussion

### Cytotoxicity against the D24 melanoma cells varies among the *C. nutans* leaves obtained from different locations

This study is the first to show variation in the cytotoxicity among the crude MeOH extracts of *C. nutans* leaves collected from 11 different locations in Malaysia, Thailand and Vietnam, with diverse geographical and climatic conditions, against the D24 melanoma cells. Current findings suggest that the geographical origin of *C. nutans* may not be a major determinant for its cytotoxicity, but differences in elevation and climatic conditions within a geographical area may contribute to the variation. Elevation and annual mean temperature significantly affected the bioactivity of *C. nutans* leaves, suggesting that samples from higher elevations and cooler climates are likely to have a higher cytotoxic effect against the D24 cells than from samples collected from lower elevations and warmer air temperature (Additional file 2: Figure S1).

It has been proposed that environmental factors that influence growing conditions are important because they can interfere with the metabolic pathways in plants and therefore on the total concentration of bioactive compounds, which in turn affects the extent of their bioactivities [29, 30]. The results of this study are in accordance with previous research, reporting variations in the levels of cytotoxicity of samples of different geographical origins with varying environmental conditions. Ayob et al. [31] reported that the crude methanol extracts of *Justicia gendarussa* (Acanthaceae) leaves from five different locations in Malaysia had different cytotoxic effects on MDA-MB-231 and −468 breast cancer cell lines. In another study, Basar et al. [32] observed considerable variation in the cytotoxicity of methanol root extracts of *Glycyrrhiza glabra* L. (Fabaceae) samples from nine different countries, which were tested against immortal human keratinocyte (HaCaT), lung adenocarcinoma (A549) and liver hepatocellular carcinoma (HepG2) cell lines.

### The crude methanol *C. nutans* leaf extract exhibits selective cytotoxicity against the D24 melanoma cells

The crude MeOH extract of *C. nutans* showed significant cytotoxicity against the D24 melanoma, but not the NHDF cells, which suggests that the extract is selective against cancer cells but not normal cells. The selective cytotoxic effect may be due to the genetic, molecular and biochemical differences in the mitochondria of cancer and normal cells [33], in this case, D24 and NHDF cells, respectively. Mitochondria generate energy that is needed by the cells in the form of ATP and they are significantly involved in the regulation of apoptosis in the cells [33]. Cancer cells, in general, have increased metabolic rates compared to their non-tumorous counterparts [34], which may be related to changes in the mitochondrial TCA cycle [35]. This altered metabolism may cause tumour mitochondria to be unstable [36] and therefore, making these cells more sensitive to the *C. nutans* crude MeOH extract. However, further studies on the molecular pathways are recommended to gain an insight into the differential effects of the crude MeOH leaf extract of *C. nutans* has on the cell death pathways in these cells.

Results of this study are in agreement with previous findings reporting that *C. nutans* leaves do have anticancer properties, although different extract and cancer cell line were used in the current study. Yong et al. [15] tested three crude leaf extracts (chloroform, methanol and aqueous) on different human cancer cell lines i.e. HepG2, neuroblastoma (IMR-32), lung (NCI-23), gastric (SNU-1), colon adenocarcinoma (LS-174 T), HeLa, K-562, and Raji, and found that chloroform extract had the highest antiproliferative effect against the latter two cell lines. Another study by Arullappan et al. [16] tested three crude leaf extracts of *C. nutans* (petroleum ether, ethyl acetate and methanol) on HeLa and K-562 cells, and found that petroleum ether extract had the strongest cytotoxic activity against both cell lines. The cytotoxicity of the *C. nutans* crude leaf extracts may be due to the presence of flavonoids, such as *C*-glycosyl flavones, which have been shown in *Mimosa pudica* (Fabaceae) [37] and *Isodon lophanthoides* var. *gerardianus* (Lamiaceae) [38] to have inhibitory effects on the proliferation of cancer cells. Further work, including bioassay-guided fractionation of the crude leaf extracts, purification and isolation of the bioactive compounds, is necessary to verify the specific compounds responsible for the activity.

### The crude methanol *C. nutans* leaf extract induces apoptosis in the D24 melanoma cells

This study is the first to examine the modes of cell death in the D24 melanoma cells treated with the crude MeOH extract in vitro. Muse cytofluorometric analysis using the characterisation of biochemical features (Annexin V to detect apoptotic cells with expressed phosphatidylserine externalisation on the cell surface and 7-AAD to distinguish dead cells) revealed a significant increase in the percentage of late apoptotic/necrotic and a significant decrease in viable cell populations, particularly at the highest test concentration (2 mg/mL) and longest treatment time (72 h), compared to the untreated controls. Besides, visual assessment of the confocal images of the Annexin V/PI double-stained D24 cells treated with the leaf extract confirmed the presence of late apoptotic/necrotic cells, although a few early apoptotic cells were observed at the lowest test concentration. Therefore, results of this study suggest that the extract induced late apoptotic/necrotic cell death in the D24 cells in a dose- and time-dependent manner.

Although utilisation of Annexin V, 7-AAD and PI is a standard procedure to observe the progression of apoptosis, this method is incapable of distinguishing between late apoptotic and primary necrotic cells, since both groups were positive to Annexin V and 7-AAD/PI [39], as shown in the Muse cytofluorometric and confocal analyses results. Therefore, to further determine the mode of cell death in D24 induced by the crude MeOH *C. nutans* extract, detection of changes in cell morphology using other types of microscopy techniques, including phase-contrast and transmission electron were used.

Optical and electron microscopy have been used to detect morphological changes that occur during apoptosis, although the latter gives better definition of subcellular changes [40]. Phase contrast images and electron micrographs of the treated D24 cells revealed classical morphological features of apoptosis, including cell shrinkage as a result of condensation of organelles and the density of cytoplasm; chromatin condensation peripherally at the nuclear membrane; fragmented and/or lobulated nucleus; and extensive blebbing of plasma membrane, budding into apoptotic bodies consisting of cytoplasm with tightly packed organelles with or without nuclear fragments [40–43]. Distinguishing apoptosis from necrosis is difficult most of the time, especially using conventional histology, and both events can happen simultaneously depending on factors like the concentration and exposure time of stimulus, the degree of ATP depletion and the availability of caspases [44]. Nevertheless, some of the typical features of necrosis include cell swelling, highly vacuolated cytoplasm and disrupted cell membrane that becomes permeable, resulting in the release of cellular contents [40, 42]. However, the treated D24 cells did not show the necrosis features when observed under a transmission electron microscope and hence, results of this study may suggest that apoptosis as the most likely type of cell death in the D24 cells, although the compound or component of the crude MeOH leaf extract of *C. nutans* that induced apoptosis is unknown. Further studies are necessary to confirm that the crude MeOH extract activates the apoptotic pathway in these cells.

Several medicinal plant species of the family Acanthaceae have been reported to induce apoptotic cell death in different cancer cell lines. For example, *Justicia spicigera* (Acanthaceae) leaves have shown to induce apoptosis in mouse fibroblasts (3 T3), human cervical carcinoma (CALO and INBL) [45] and HeLa [46] cells, while crude leaf extracts of *Ruellia tuberosa* (Acanthaceae) and *Andrographis paniculata* (Acanthaceae) exhibited potent apoptogenic activity on HepG2 [47] and human oropharyngeal cancer cells (KB) [48] cells, respectively. It has been described that a synergistic activity of interferon (IFN)-γ and tumour necrosis factor (TNF)-α strongly induces apoptosis in HaCaT keratinocyte cells [49]. However, Thongrakard and Tencomnao [50] showed that the crude ethanol *C. nutans* leaf extract significantly inhibited the induction of apoptosis by IFN-γ/TNF-α in HaCaT cells. Nonetheless, it should be noted that the stereotype outcome either as apoptosis or necrosis cannot always be expected. This is because induction or inhibition of cell death modes depends on a number of factors, such as the plant species, preparation methods (crude extracts, fractions and isolated compounds), concentrations and exposure durations of stimuli, cell types and the nature of the cell death signal [44, 51–53].

## Conclusions

In conclusion, it was demonstrated that the crude MeOH leaf extract of *C. nutans* was cytotoxic to D24 melanoma cells but was less harmful to normal fibroblasts. The present study also showed that collection sites with different environmental factors can affect the bioactivity of *C. nutans* leaves, where the leaves of plants grown at higher elevations and lower air temperatures had higher levels of cytotoxicity than those grown at lower elevations and higher air temperatures. The crude extract also induced apoptotic cell death in the D24 cells in a dose- and time-dependent manner. These observations suggest that the crude MeOH *C. nutans* leaf extract can be used to supplement current regimens used for cancer prevention or treatment. The results are useful for the development of *C. nutans* as a potential chemotherapeutic agent for the treatment of skin melanoma and other cancers.

However, further work is needed to identify the active compound or component in the crude MeOH *C. nutans* leaf extract and to determine their anticancer efficacies. Additionally, further research to understand the underlying mechanism in the induced cell death, especially the cellular signalling pathways involved and in vivo testing of the observed anticancer activity are essential to unveil the full potential use of *C. nutans* in cancer therapy.

## Additional files

Additional file 1: Table S1. The total phenolic (TPC) and flavonoid (TFC) content (mean mg GAE or QE/g dry extract ± SD, *n* = 3) of the different crude extracts of *C. nutans* leaves. (DOCX 121 kb) Additional file 2: Figure S1. Scatter plots showing correlations between cytotoxicity of the crude MeOH leaf extracts of 11 *C. nutans* samples and different environmental factors, including elevation (a) and annual mean temperature (b). * *p* ≤ 0.05. (JPG 55 kb)

## Abbreviations

7-AAD: 7-aminoactinomycin D
ANOVA: Analysis of variance
CCK-8: Cell counting kit-8
DMEM: Dulbecco’s modified eagle medium
DMSO: Dimethyl sulfoxide
EC_50_: Half maximal effective concentration
FBS: Foetal bovine serum
FITC: Fluorescein isothiocyanate
MeOH: Methanol
PI: Propidium iodide
RPMI: Roswell Park Memorial Institute medium
SD: Standard deviation
TEM: Transmission electron microscopy

## References

[1] Forman D, Ferlay J, Stewart BW, Wild CP (2014). The global and regional burden of cancer. World cancer report 2014.

[2] Uong A, Zon LI (2010). Melanocytes in development and cancer. J Cell Physiol.

[3] Cifola I, Pietrelli A, Consolandi C, Severgnini M, Mangano E, Russo V, Bellis GD, Battaglia C (2013). Comprehensive genomic characterization of cutaneous malignant melanoma cell lines derived from metastatic lesions by whole-exome sequencing and SNP array profiling. PLoS ONE.

[4] Ferlay J, Soerjomataram I, Ervik M, Dikshit R, Eser S, Mathers C, Rebelo M, Parkin DM, Forman D, Bray F. GLOBOCAN 2012 v1.0, Cancer Incidence and Mortality Worldwide: IARC CancerBase No. 11 International Agency for Research on Cancer, Lyon. 2013. http://globocan.iarc.fr/. Accessed 27 June 2015.

[5] Batus M, Waheed S, Ruby C, Petersen L, Bines S, Kaufman H (2013). Optimal management of metastatic melanoma: current strategies and future directions. Am J Clin Dermatol.

[6] Bhatia S, Tykodi SS, Thompson JA (2009). Treatment of metastatic melanoma: an overview. Oncology.

[7] Iyer AK, Singh A, Ganta S, Amiji MM (2013). Role of integrated cancer nanomedicine in overcoming drug resistance. Adv Drug Deliv Rev.

[8] Kunjachan S, Rychlik B, Storm G, Kiessling F, Lammers T (2013). Multidrug resistance: physiological principles and nanomedical solutions. Adv Drug Deliv Rev.

[9] Markman JL, Rekechenetskiy A, Holler E, Ljubimova JY (2013). Nanomedicine therapeutic approaches to overcome cancer drug resistance. Adv Drug Deliv Rev.

[10] Pratheeshkumar P, Sreekala C, Zhang Z, Budhraja A, Ding S, Son YO, Wang X, Hitron A, Hyun-Jung K, Wang L, Lee JC, Shi X (2012). Cancer prevention with promising natural products: mechanisms of action and molecular targets. Anticancer Agents Med Chem.

[11] Johnson IT (2007). Phytochemicals and cancer. Proc Nutr Soc.

[12] Chinembiri TN, du Plessis LH, Gerber M, Hamman JH, du Plessis J (2014). Review of natural compounds for potential skin cancer treatment. Molecules.

[13] Wanikiat P, Panthong A, Sujayanon P, Yoosook C, Rossi AG, Reutrakul V (2008). The anti-inflammatory effects and the inhibition of neutrophil responsiveness by *Barleria lupulina* and *Clinacanthus nutans* extracts. J Ethnopharmacol.

[14] Pannangpetch P, Laupattarakasem P, Kukongviriyapan V, Kukongviriyapan U, Kongyingyoes B, Aromdee C (2007). Antioxidant activity and protective effect against oxidative hemolysis of *Clinacanthus nutans* (Burm. F.) Lindau. Songklanakarin J Sci Technol.

[15] Yong YK, Tan JJ, Teh SS, Mah SH, Ee GCL, Chiong HS, Ahmad Z (2013). *Clinacanthus nutans* extracts are antioxidant with antiproliferative affect on cultured human cancer cell lines. Evid Based Complement Alternat Med.

[16] Arullappan S, Rajamanickam P, Thevar N, Kodimani CC (2014). In vitro screening of cytotoxic, antimicrobial and antioxidant activities of *Clinacanthus nutans* (Acanthaceae) leaf extracts. Trop J Pharm Res.

[17] Tiew WP, P'ng XW, Chin JH, Akowuah GA (2014). Effect of methanol extract of *Clinacanthus nutans* on serum biochemical parameters in rats. J Appl Pharm.

[18] Wong FC, Yong AL, Ting EPS, Khoo SC, Ong HC, Chai TT (2014). Antioxidant, metal chelating, anti-glucosidase activities and phytochemical analysis of selected tropical medicinal plants. Iranian J Pharm Res.

[19] Thongchai S, Ekalaksananan T, Pientong C, Aromdee C, Seubsasana S, Sukpol C, Kongyingyoes B (2008). Anti-herpes simplex virus type 1 activity of crude ethyl acetate extract derived from leaves of *Clinacanthus nutans* Lindau. J Sci Technol Mahasarakham U.

[20] Sakdarat S, Shuyprom A, Pientong C, Ekalaksananan T, Thongchai S (2009). Bioactive constituents from the leaves of *Clinacanthus nutans* Lindau. Bioorg Med Chem.

[21] Kunsorn P, Ruangrungsi N, Lipipun V, Khanboon A, Rungsihirunrat K (2013). The identities and anti-herpes simplex virus activity of *Clinacanthus nutans* and *Clinacanthus siamensis*. Asian Pac J Trop Biomed.

[22] Vachirayonstien T, Promkhatkaew D, Bunjob M, Chueyprom A, Chavalittumrong P, Sawanpanyalert P (2010). Molecular evaluation of extracellular activity of medicinal herb *Clinacanthus nutans* against herpes simplex virus type-2. Nat Prod Res.

[23] Thawaranantha D, Balachandra K, Jongtrakulsiri S, Chavalittumrong P, Bhumiswasdi J, Janyavasu C (1992). In vitro antiviral activity of *Clinacanthus nutans* on varicellazoster virus. Siriraj Hosp Gaz.

[24] Sookmai W, Ekalaksananan T, Pientong C, Sakdarat S, Kongyingyoes B (2011). The anti-papillomavirus infectivity of *Clinacanthus nutans* compounds. Srinagarind Med J.

[25] Tu SF, Liu RH, Cheng YB, Hsu YM, Du YC, El-Shazly M, Wu YC, Chang FR (2014). Chemical constituents and bioactivities of *Clinacanthus nutans* aerial parts. Molecules.

[26] Hijmans RJ, Guarino L, Mathur P. DIVA-GIS. 7.5 ed. University of California Davis, California; 2012.

[27] Gatan. Gatan Microscopy Suite (2013). DigitalMicrograph. 2.3 ed.

[28] Minitab (2013). Minitab statistical software. 17 ed.

[29] Akula R, Ravishankar GA (2011). Influence of abiotic stress signals on secondary metabolites in plants. Plant Signal Behav.

[30] Radušienė J, Karpavičienė B, Stanius Ž (2012). Effect of external and internal factors on secondary metabolites accumulation in St. John’s worth. Bot Lith.

[31] Ayob Z, Mohd Bohari SP, Abd Samad A, Jamil S (2014). Cytotoxic activities against breast cancer cells of local *Justicia gendarussa* crude extracts. Evid Based Complement Alternat Med.

[32] Basar N, Oridupa OA, Ritchie KJ, Nahar L, Osman NM, Stafford A, Kushiev H, Kan A, Sarker SD (2015). Comparative cytotoxicity of *Glycyrrhiza glabra* roots from different geographical origins against immortal human keratinocyte (HaCaT), lung adenocarcinoma (A549) and liver carcinoma (HepG2) cells. Phytother Res.

[33] McLachlan A, Kekre N, McNulty J, Pandey S (2005). Pancratistatin: a natural anti-cancer compound that targets mitochondria specifically in cancer cells to induce apoptosis. Apoptosis.

[34] Rodrigues MF, Obre E, de Melo FH, Santos GC, Galina A, Jasiulionis MG, Rossignol R, Rumjanek FD, Amoedo ND (2016). Enhanced OXPHOS, glutaminolysis and beta-oxidation constitute the metastatic phenotype of melanoma cells. Biochem J.

[35] Piva TJ, McEvoy-Bowe E (1998). Oxidation of glutamine in HeLa cells: role and control of truncated TCA cycles in tumour mitochondria. J Cell Biochem.

[36] Liu T, Hannafon B, Gill L, Kelly W, Benbrook D (2007). Flex-Hets differentially induce apoptosis in cancer over normal cells by directly targeting mitochondria. Mol Cancer Ther.

[37] Li YX, Zhu JX, Yang HX, Yuan K (2011). Studies on antitumor activities of six glycosylflavones from *Mimosa pudica*. 2011 International Symposium on IT in Medicine and Education (ITME 2011); 2011 9–11.

[38] Zhang Y, Tang H, Li A, Xu L, Chen J, Huang S, He L (2015). Determination of six C-glycoside flavones and antitumor activity of water-soluble total flavonoids from *Isodon lophanthoides* var. *gerardianus*. Zhongguo Zhong Yao Za Zhi.

[39] Brauchle E, Thude S, Brucker SY, Schenke-Layland K (2014). Cell death stages in single apoptotic and necrotic cells monitored by Raman microspectroscopy. Sci Rep.

[40] Elmore S (2007). Apoptosis: a review of programmed cell death. Toxicol Pathol.

[41] Chen Y, Sun R, Han W, Zhang Y, Song Q, Di C, Ma D (2001). Nuclear translocation of PDCD5 (TFAR19): an early signal for apoptosis?. FEBS Lett.

[42] Ziegler U, Groscurth P (2004). Morphological features of cell death. News Physiol Sci.

[43] Svejda B, Aguiriano-Moser V, Sturm S, Hoger H, Ingolic E, Siegl V, Stuppner H, Pfragner R (2010). Anticancer activity of novel plant extracts from *Trailliaedoxa gracilis* (W. W. Smith & Forrest) in human carcinoid KRJ-I cells. Anticancer Res.

[44] Zeiss CJ (2003). The apoptosis-necrosis continuum: insights from genetically altered mice. Vet Pathol.

[45] Cáceres-Cortés JR, Cantú-Garza FA, Mendoza-Mata MT, Chavez-González MA, Ramos-Mandujano G, Zambrano-Ramírez IR (2001). Cytotoxic activity of *Justicia spicigera* is inhibited by bcl-2 proto-oncogene and induces apoptosis in a cell cycle dependent fashion. Phytother Res.

[46] Alonso-Castro AJ, Ortiz-Sánchez E, Domínguez F, Arana-Argáez V, Juárez-Vázquez MC, Chávez M, Carranza-Álvarez C, Gaspar-Ramírez O, Espinosa-Reyes G, López-Toledo G, Ortiz-Andrade R, García-Carrancá A (2012). Antitumor and immunomodulatory effects of *Justicia spicigera* Schltdl (Acanthaceae). J Ethnopharmacol.

[47] Dey S, Roy S, Deb N, Sen KK, Besra SE (2013). Anti-carcinogenic activity of *Ruellia tuberosa* L. (Acanthaceae) leaf extract on hepatoma cell line & increased superoxide dismutase activity on macrophage cell lysate. Int J Pharm Pharm Sci.

[48] Saengkhae C, Meewassanasuk K (2009). Antiproliferation and apoptosis of the crude extract of *Andrographis paniculata* Nees, on human oropharyngeal cancer cells (KB) in vitro. Thai J Physiol Sci.

[49] Konur A, Schulz U, Eissner G, Andreesen R, Holler E (2005). Interferon (IFN)-gamma is a main mediator of keratinocyte (HaCaT) apoptosis and contributes to autocrine IFN-gamma and tumour necrosis factor-alpha production. Br J Dermatol.

[50] Thongrakard V, Tencomnao T (2010). Modulatory effects of Thai medicinal plant extract on proinflammatory cytokines-induced apoptosis in human keratinocyte HaCaT cells. Afr J Biotechnol.

[51] Hirsch T, Marchetti P, Susin SA, Dallaporta B, Zamzami N, Marzo I, Geuskens M, Kroemer G (1997). The apoptosis-necrosis paradox. Apoptogenic proteases activated after mitochondrial permeability transition determine the mode of cell death. Oncogene.

[52] Ahmad NH, Rahim RA, Mat I (2010). *Catharanthus roseus* aqueous extract is cytotoxic to jurkat leukaemic T-cells but induces the proliferation of normal peripheral blood mononuclear cells. Trop Life Sci Res.

[53] Cock IE (2011). Problems of reproducibility and efficacy of bioassays using crude extratcs, with reference to *Aloe vera*. Phcog Commn.

