# Supplementary material for: Comparison of cytotoxicity between extracts of Clinacanthus nutans (Burm. f.) Lindau leaves from different locations and the induction of apoptosis by the crude methanol leaf extract in D24 human melanoma cells
Source: BMC Complement Altern Med. 2016 Sep 20;16:368. doi: 10.1186/s12906-016-1348-x (PMC5029048; doi:10.1186/s12906-016-1348-x)
Supplement: Additional file 1: Table S1. — The total phenolic (TPC) and flavonoid (TFC) content (mean mg GAE or QE/g dry extract ± SD, n = 3) of the different crude extracts of C. nutans leaves. (DOCX 121 kb) [file 12906_2016_1348_MOESM1_ESM.docx]

**Table S1** The total phenolic (TPC) and flavonoid (TFC) content (mean mg GAE or QE/g dry extract ± SD, n = 3) of the different crude extracts of *C. nutans* leaves.

| **Extract** | **Crude extract yield (% w/w)** | **TPC** | | | **TFC** | | |
| --- | --- | --- | --- | --- | --- | --- | --- |
| Dichloromethane | 19.16 | 36.68 | ± | 0.04^b^ | 8.48 | ± | 3.14^A^ |
| Ethanol | 6.53 | 24.57 | ± | 0.07^c^ | 7.09 | ± | 2.98^A^ |
| Methanol | 7.87 | 45.04 | ± | 0.06^a^ | 12.17 | ± | 4.52^A^ |

Note: Samples that do not share a letter (lower and upper cases) are significantly different at p ≤ 0.05 level.
