# Supplementary figures and images for: Comparison of cytotoxicity between extracts of Clinacanthus nutans (Burm. f.) Lindau leaves from different locations and the induction of apoptosis by the crude methanol leaf extract in D24 human melanoma cells
Source: BMC Complement Altern Med. 2016 Sep 20;16:368. doi: 10.1186/s12906-016-1348-x (PMC5029048; doi:10.1186/s12906-016-1348-x)

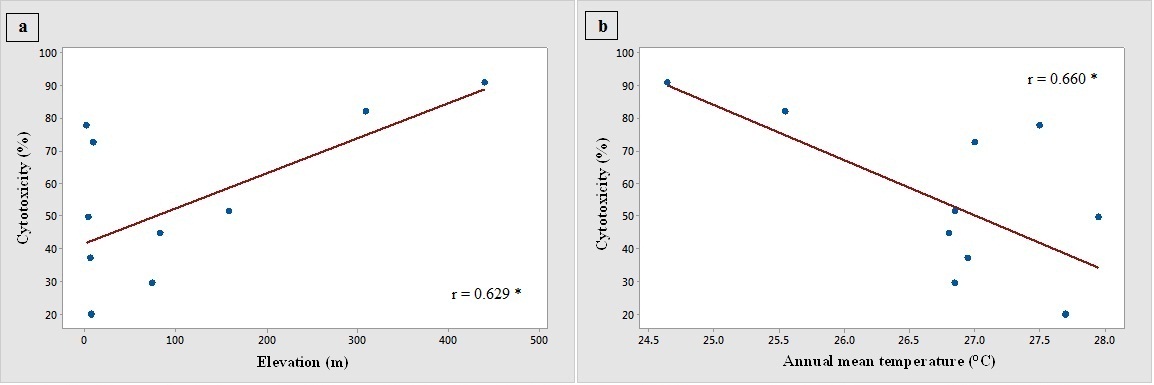

Supplement: Additional file 2: Figure S1. — Scatter plots showing correlations between cytotoxicity of the crude MeOH leaf extracts of 11 C. nutans samples and different environmental factors, including elevation (a) and annual mean temperature (b). * p ≤ 0.05. (JPG 55 kb) [file 12906_2016_1348_MOESM2_ESM.jpg]
